# Importance of validating antibody panels: Anti-PD-L1 clone binds AF700 fluorophore

**DOI:** 10.1016/j.jim.2020.112795

**Published:** 2020-08

**Authors:** Michael J. Hughes, Helen M. McGettrick, Elizabeth Sapey

**Affiliations:** a Birmingham Acute Care Research, Institute of Inflammation and Ageing, College of Medical and Dental Sciences, University of Birmingham, Birmingham B15 2TT, UK; b Rheumatology Research Group, Institute of Inflammation and Ageing, University of Birmingham, Edgbaston, Birmingham B15 2GW, UK

**Keywords:** 7AAD 7, aminoactinomycin D, AF, Alexa Fluor™, AnV, Annexin V (5), APC, Allophycocyanin, BV, Brilliant Violet™, BSA, Bovine Serum Albumin, CD, Cluster of Differentiation, COPD, Chronic Obstructive Pulmonary Disease, FcγRIII, Fragment-crystallisable Gamma Receptor Three, FEV_1_, Forced Expiratory Volume in 1 s, FITC, Fluorescein isothiocyanate, FSC, Forward Scatter, FVC, Forced Vital Capacity, GOLD, Global Initiative for Chronic Obstructive Lung Disease, MFI, Median Fluorescence Intensity, NaCl, Sodium Chloride, PBMC, Peripheral Blood Mononuclear Cell, PBS, Phosphate Buffered Saline, PD-1, Programmed Death receptor protein 1, PD-L1, Programmed Death receptor ligand 1, PD-L2, Programmed Death receptor ligand 2, Pen/Strep, Penicillin/Streptomycin, RPMI, Roswell Park Memorial Institute, SLE, Systemic Lupus Erythematosus, SSC, Side Scatter, STAT, Signal Transducer and Activation of Transcription

## Abstract

Researchers routinely use antibodies to assess the expression levels of proteins on the surface or intracellularly in a variety of different cell types. In this current study we highlight the importance of careful validation of antibodies for analysis of protein expression by flow cytometry and how failure to do so can significantly impact the interpretation of the data generated leading to false-positive results. There has been increasing awareness of the role the programmed death receptor 1 (PD-1) pathway plays in health and disease and a potential that programme death ligand 1 (PD-L1) may play a role in inflammatory disease. We aimed to investigate PD-L1 expression on human neutrophils isolated from healthy individuals and patients diagnosed with chronic obstructive pulmonary disease (COPD). We observed an increase in surface expression of PD-L1 by human neutrophils when incubated with AlexaFluor™700-conjugated anti-CD16. Through careful interrogation and antibody validation, we found a novel interaction between a commercially available anti-PD-L1 antibody and the AlexaFluor™700 fluorophore, resulting in this observed increase in PD-L1 signal. Surface expression of PD-L1 was not observed on neutrophils from healthy volunteers or patients with COPD when clone 29E.2A3 of anti-PD-L1 was not used with AlexaFluor™700-conjugated anti-CD16. This highlights the importance of robust antibody validation to ensure antibody compatibility in the context of multi-parametric flow cytometry panels. We also show that, without these validation experiments, novel neutrophil phenotypes could be falsely reported – an important consideration when there is increasing interest in neutrophil heterogeneity.

## 1 Introduction

Neutrophils are the most abundant immune cell commonly implicated in a number of chronic inflammatory diseases, such as chronic obstructive pulmonary disease (COPD), and make up around 40% of circulating immune cells in health (Summers et al., 2010). There is a growing body of research focusing on identifying and studying neutrophil phenotypes and smaller subpopulations (Hellebrekers et al., 2018; Hughes et al., 2019). In all cases, flow cytometry remains the gold standard tool for interrogating surface expression of a variety of molecules. Data obtained from this methodology is only as good as the antibody validation protocols researchers undertake at the start of every study and the pitfalls of commercial antibodies has been pointed out previously (Bordeaux et al., 2010), specifically using immunohistochemistry (Jositsch et al., 2009). The importance remains with flow cytometry to ensure that fluorescent signals indicate expression of the protein of interest, and not background or non-specific staining of the cells.

Programmed Death receptor protein 1 (PD-1) has been the focus of multiple cancer immunotherapies (Rotte, 2019) and research into the role of PD-1 in cancer therapy was awarded with a Nobel Prize in 2018 (Rotte et al., 2018). PD-1 is predominantly expressed on T cells and engagement of PD-1 inhibits T cell proliferation and activation, maintaining immune tolerance in health (Keir et al., 2008; Wei et al., 2013), and has been implicated in evasion of immune-medicated clearance of cancer cells (Dong et al., 2015). The PD-1 receptor has two natural ligands, PD-L1 and PD-L2. PD-L1 expression by tumour cells inhibits T cell-mediated killing, and there is an evolving clinical programme to block PD-L1 as a therapeutic in cancer therapy (Chen et al., 2016; Hahn et al., 2017; Huang et al., 2015). Less is known about PD-L2; its expression is less ubiquitous than PD-L1 (Dail et al., 2016) but evidence suggests PD-L2 also inhibits T-cell function (Latchman et al., 2001). It is clear that the PD-1 axis has a role to play in cancer, but the importance of this axis may extend to other disease areas and cell types.

Far less is known about the expression of PD-L1 on neutrophils and its importance in neutrophil function in health and disease. That said, neutrophil PD-L1 may be of interest in inflammatory diseases such as COPD where neutrophils are abundant, but bacterial clearance appears reduced (Hodge and Reynolds, 2012) and it has been postulated that PD-L1 is reduced on macrophage and dendritic cells in COPD, rather than increased as in cancer (Stoll et al., 2016). Neutrophil PD-L1 expression has been implicated in other inflammatory conditions. For example, an increased proportion of PD-L1-expressing neutrophils have been detected in the blood of systemic lupus erythematous patients, and this increase positively correlated with disease severity (Luo et al., 2016). Moreover, human neutrophils up-regulated PD-L1 expression when exposed to conditioned media from cancer associated fibroblasts in vitro leading to a reduction in T cell proliferation and increased neutrophil survival (Cheng et al., 2018). Furthermore, isolated peripheral neutrophils from patients with active tuberculosis infection have been reported to have elevated levels of PD-L1 surface expression compared to healthy volunteers (McNab et al., 2011). Thus, current evidence suggests that neutrophils can express PD-L1 during infection and chronic inflammatory disease, and that this heightened expression may play a role in disease. However, the impact of altering the PD-1/PD-L1 axis in chronic inflammatory lung disease is not yet understood, especially if PD-L1 expression may be reduced in the lung of patients with COPD (Stoll et al., 2016).

In this work, we aimed to investigate the expression of PD-L1 by neutrophils from patients with COPD and healthy volunteers, hypothesising that neutrophils from patients with COPD would have higher PD-L1 expression. As part of a larger panel of antibodies, anti-PD-L1 and anti-CD16 (one of the most common ways to identify neutrophils in flow cytometry) were validated for the identification of surface PD-L1 expression on human peripheral blood neutrophils. Incubation with both anti-CD16 and anti-PD-L1 resulted in observed PD-L1 expression by human neutrophils. Expression of PD-L1 was not observed in the absence of anti-CD16, or with specific clones. Through careful validation, we found that a specific clone of anti-PD-L1 was capable of specifically binding the AF700 fluorophore, resulting in false-positive detection of PD-L1 expression. This novel cross-reactivity highlights the importance of rigorous validation of antibody combinations to prevent false-positive discoveries.

## 2 Methods

### 2.1 Donor consent

This study was conducted in accordance with the Declaration of Helsinki. All human blood samples were collected following ethical approval (West Midlands – Solihull Research Ethics Committee, Reference 18/WM/0097) and informed consent was provided by all participants. Healthy volunteers were recruited from the Institute of Inflammation and Ageing, University of Birmingham and through the Birmingham 1000 Elders Cohort. Patients with COPD, defined according to GOLD 2019 guidelines with a ratio of forced expiratory volume in 1 s (FEV_1_) to forced vital capacity (FVC) of less than 0.7 (GOLD, 2019), were recruited through the Chronic Disease Resource Centre at the Centre for Translational Inflammation Research, Queen Elizabeth Hospital, Birmingham. Patient demographics are shown in Table 1.

**Table 1:** Participant demographics.

Group	Age (years), median (IQR)	% male	FEV_1_ (% predicted), median (IQR)	FVC (% predicted), median (IQR)	Pack year history, median (IQR)
Heathy young	27 (4)	50	N/A	N/A	N/A
Healthy elderly	N/A	50*	N/A	N/A	N/A
COPD	77.5 (7.25)	83	61.5 (29)	97.5 (16)	40 (33)

Data given as median (interquartile range) where appropriate. Abbreviations: forced expiratory volume in one second, FEV_1_; forced vital capacity, FVC; not available, N/A. *Data only available for 2 participants

### 2.2 Isolation of neutrophils from peripheral blood

Human blood was collected using the vacutainer system (BD Biosciences, Wokingham, UK) into heparin containing tubes via venepuncture. Neutrophils were isolated as previously described (Jepsen and Skottun, 1982). Briefly, whole blood was mixed 6:1 with dextran (2% w/v; Sigma-Aldrich, Poole, UK) in 0.154 M saline for 30 min. Isotonic Percoll was made using 9:1 *v*/v Percoll (Sigma-Aldrich) to sterile NaCl (1.54 M) and the remaining white cell fraction was added to a discontinuous Percoll gradient (56% and 80% v/v of isotonic Percoll in 0.154 M saline). The gradient was centrifuged at 470 *g* for 20 min without acceleration or brake. The top plasma and cell layer were removed and discarded, and then the lower granulocyte band obtained and washed in phosphate buffered saline (PBS; Sigma-Aldrich) before resuspension in Roswell Park Memorial Institute 1640 media containing 2 mM l-glutamine (RPMI; ThermoFisher Scientific, Loughborough, UK) supplemented with Penicillin/Streptomycin (Pen/Strep; 1% *w*/w; Sigma-Aldrich) to a final concentration of 1 × 10^6^ cells/mL.

### 2.3 Isolation of mononuclear cells

Human blood was collected using the vacutainer system into heparin containing tubes via venepuncture. Monocytes from healthy volunteer blood were isolated using a two-step gradient of Histopaque-1077 layered on top of Histopaque-1119 (Sigma-Aldrich) as previously described (McGettrick et al., 2010). Cells were then washed once in PBS and resuspended in RPMI containing 2 mM l-glutamine supplemented with Pen/Strep (1% *v*/v), foetal bovine serum (20% v/v; Sigma-Aldrich) and 10 μg/mL of phytohemagglutinin (Sigma-Aldrich) to stimulate PD-L1 expression (Campbell et al., 2009). Monocytes were cultured for 3 days at 37 °C 5% CO_2_. Cells were detached using TripLE Express (ThermoFisher Scientific) and washed in PBS containing 2% Bovine Serum Albumin (BSA; Sigma-Aldrich) and diluted to 1 × 10^6^ cells/mL.

### 2.4 Antibody incubation

Neutrophils or monocytes (1 × 10^6^ cells/mL) were incubated for 20 min on ice with flurochrome-conjugated antibodies (Table 2). Cells were washed once in PBS containing BSA (2% w/v;) and once in Annexin V Staining Buffer (AnV Buffer; BioLegend, London, UK). Cells were then incubated at room temperature in AnV Buffer with 0.06 μg/mL annexin V (1:40; BioLegend, UK) for 15 min in the dark before washing once with Annexin V staining buffer. Cells were resuspended in AnV buffer containing 2.5 μg/mL 7-aminoactinomycin D (7AAD; BioLegend) dye immediately prior to flow cytometry.

**Table 2:** List of antibodies.

Target	Fluorophore	Manufacturer	Clone	Standard dilution
CD16	AF700	BioLegend	3G8	1:100
	AF700	Invitrogen	3G8	1:20
	AF700	eBiosciences	eBioCB16	1:100
	FITC	eBiosciences	eBioCB16	1:100
	FITC	Miltenyi Biotec	VEP-13	1:100
PD-L1	BV605	BioLegend	29E.2A3	1:100
	APC	R&D Systems	130,021	1:100
IgG1κ	AF700	BioLegend	MOPC-21	3:200
	FITC	BioLegend	MOPC-21	1:100
	APC	BioLegend	MOPC-21	1:100
IgG2bκ	BV605	BioLegend	MPC-11	3:200
CD66b	AF700	BioLegend	G10F5	1:100

Antibodies grouped by target. The dilution stated are those used unless otherwise stated. Abbreviations: AlexaFluor™700, AF700; Fluorescein isothiocyanate, FITC; Brilliant Violet™, BV; Allophycocyanin, APC.

### 2.5 Flow cytometry

Surface expression detection was carried out by flow cytometry using a 4-laser Fortessa X20 (BD Biosciences, USA) and recorded using FACSDiva software (V8.0.1, BD Biosciences). Compensation was carried out using compensation beads (BD Biosciences), or neutrophils in the case of 7AAD, and 5000 neutrophil events were collected based on FSC/SSC properties. During antibody validation, the staining index (Maecker et al., 2004) was calculated as follows using the median fluorescence intensity (MFI):

$$\mathrm{Staining Index}=\frac{\left(\mathrm{MFI}\;\mathrm{antibody}-\mathrm{MFI}\;\mathrm{isotype control}\right)}{2\times \mathrm{robust Standard Deviation of the isotype control}}$$

### 2.6 Statistical analysis

Flow cytometry data exported from FlowJo (Version 10, BD, USA) was visualised using GraphPad Prism (Version 8.2.0 for Windows, GraphPad Software, USA). Data were expressed as median and interquartile range. Where only two groups were analysed, a Wilcoxon matched-pairs signed rank test (paired data) was performed. This test is limited as *P* < .05 can only be obtained with *n* > 5 and, in some cases, the reported *p*-value reaches the limit of this test. In all cases, a value of *p* < .05 was considered statistically significant.

## 3 Results and discussion

### 3.1 Antibody titration and validation

**Fig. 1:**
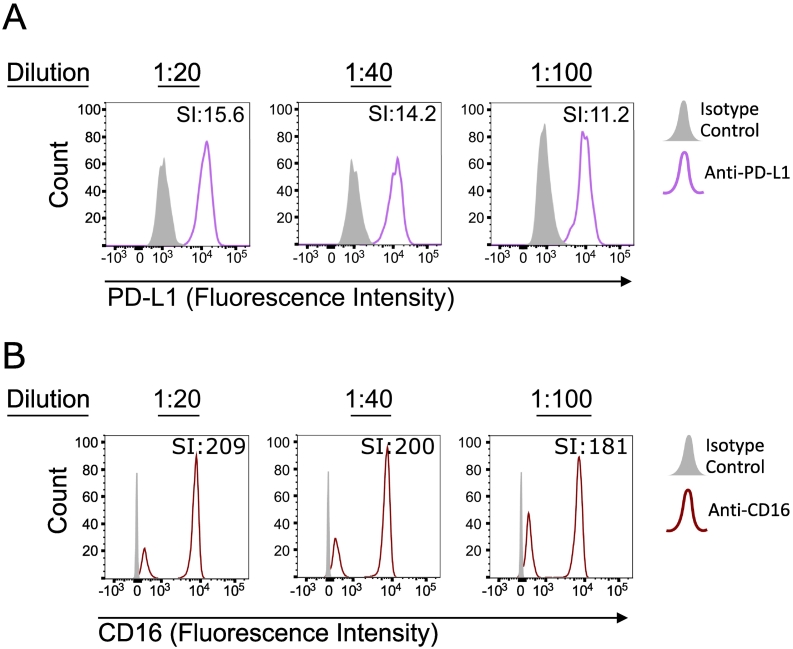
Antibody titration and target validation. A) Monocytes stimulated with 10 μg/mL of phytohemagglutinin were stained with decreasing concentrations of anti-PD-L1 (purple line; clone 29E.2A3; BioLegend) or relevant IgG isotype (grey, filled) (B) Neutrophils were stained with decreasing concentrations of anti-CD16 (red line; clone 3G8; BioLegend), or relevant IgG isotype (grey, filled). In both cases, samples were analysed by flow cytometry and a staining index (SI) was determined for each dilution. (For interpretation of the references to colour in this figure legend, the reader is referred to the web version of this article.)

As discussed, validation of each antibody used in flow cytometry is vital to provide confidence in the results obtained. A staining index (Maecker et al., 2004) can also be used to guide antibody dilution selection as it provides a quantitative measure of resolution between the positive and negative population (Fig. 1A and B). A higher staining index demonstrates a greater separation between the positive population and negative population, but this does not directly relate to absolute saturation of any given antibody. Initial validation of BV605-conjugated anti-PD-L1 (BioLegend, UK; clone 29E.2A3) was carried out on monocytes cultured for three days using three dilutions: 1:20, 1:40 and 1:100 (Fig. 1A). Similarly, AF700-conjugated anti-CD16 (BioLegend, UK; clone 3G8) was titrated at 1:20, 1:40 and 1:100 using isolated neutrophils (Fig. 1B). These titrations confirmed the ability of each antibody to specifically bind the cells of interest and good resolution between the positive and negative populations could be achieved at all antibody concentrations. Therefore, the lowest dilution of 1:100 was selected for both antibodies.

### 3.2 Basal expression of PD-L1 on neutrophils

**Fig. 2:**
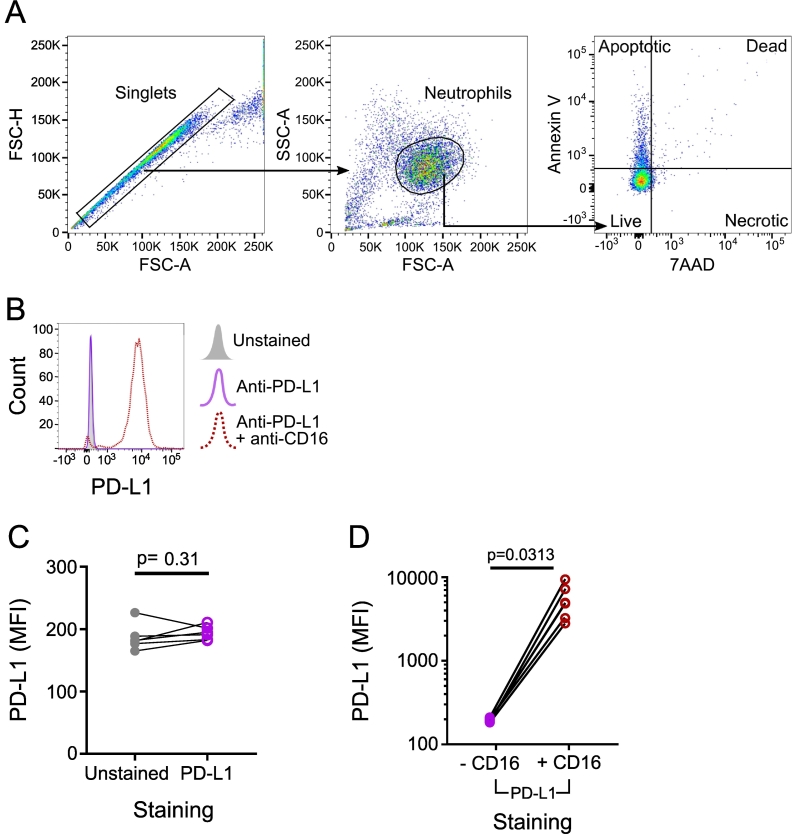
Expression of PD-L1 on human neutrophils. A) The gating strategy used to identify live neutrophil populations. Firstly, doublets were excluded based on forward scatter (FSC) area (A) vs height (H). Neutrophils were then identified by the FSC and side scatter (SSC) profile. Cell viability was assessed using annexin V and 7AAD, where neutrophils double negative for annexin V and 7AAD were considered live and non-apoptotic. This plot shows a representative example from a healthy young participant. B) A representative histogram plot of PD-L1 surface expression using unstained neutrophils (grey, filled), anti-PD-L1 stained (purple; BioLegend; Clone 29E.2A3) or both anti-PD-L1 and anti-CD16 stained (red, dotted; BioLegend; Clone 3G8) neutrophils. Analysis performed using flow cytometry and is representative of *n* = 6 experiments with n = 6 donors. C) Quantification of n = 6 donors as in B, showing the median fluorescence intensity (MFI) of PD-L1 on unstained (grey, filled) and anti-PD-L1 stained (purple, open) samples. D) Quantification of n = 6 donors as in B, showing the MFI of PD-L1 on samples stained with anti-PD-L1 and co-stained with or without anti-CD16 on a log scale. *P* values in C and D determined by Wilcoxon matched-pairs signed rank test. (For interpretation of the references to colour in this figure legend, the reader is referred to the web version of this article.)

In order to assess PD-L1 expression on neutrophils from healthy volunteers, isolated neutrophils were stained with BV605-conjugated anti-PD-L1 (BioLegend, clone 29E.2A3) and analysed using the flow cytometry gating strategy shown in Fig. 2A. The fluorescence in the stained samples is comparable to unstained controls, demonstrating no detectable expression of PD-L1 on the surface of these neutrophils (Fig. 2B and C) in line with existing literature (Cheng et al., 2018; Wang et al., 2015). As we wanted to reliably identify neutrophils, CD16 staining was incorporated using AF700-conjugated anti-CD-16 (Clone 3G8; BioLegend, UK). Unexpectedly, the addition of anti-CD16 resulted in a substantial increase in observed PD-L1 expression (Fig. 2B and D). The direct comparison of neutrophils stained with anti-PD-L1 alone and those stained with both anti-PD-L1 and anti-CD16 single-stained controls demonstrated robust and repeatable increased PD-L1 expression (Fig. 2D and Table S1).

### 3.3 Validation of spectral overlap

In order to rule out spectral overlap in our results, we checked for spill over of AF700-conjugated anti-CD16 (Clone 3G8; BioLegend) single-stained neutrophils into the BV605 channel – the detection channel for PD-L1. In CD16-positive cells, there was no detectable fluorescence signal in the BV605 channel above unstained controls (Supplementary Fig. S1), therefore demonstrating that an increase in PD-L1 detection from measurement of the BV605 median fluorescence intensity (MFI) was not due to spectral overlap. Therefore, it was highly likely the anti-PD-L1 antibody was indeed specifically binding to neutrophils and this led to the possibility that anti-CD16 was causing changes in the expression of PD-L1 on the surface of neutrophils.

### 3.4 Clone specificity of induced PD-L1 expression

It has been previously reported that PD-L1 expression can be induced on neutrophils through the Signal Transducer And Activator Of Transcription (STAT)3 pathway (Cheng et al., 2018), downstream of interleukin-6 binding. Previous studies have also shown that cross-linking CD16 leads to Ca^2+^ release and PI3K signalling in neutrophils (Chuang et al., 2000). Whilst STAT3 and PI3K signalling are distinct, these two studies support the possibility that engagement of CD16 could induce changes that may lead to increases in surface expression of PD-L1 on neutrophils.

**Fig. 3:**
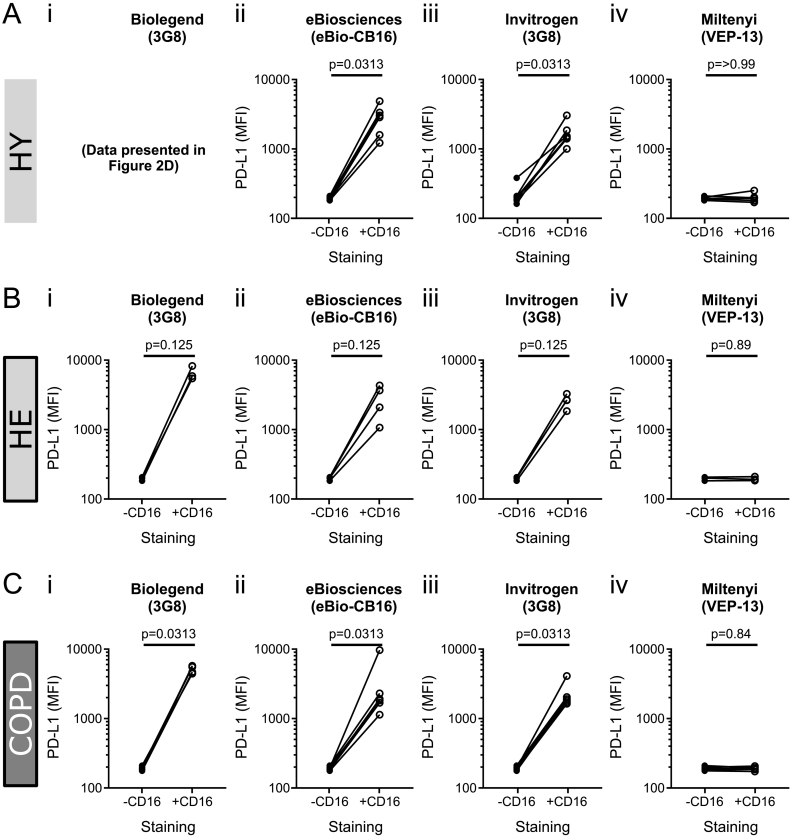
Comparison of anti-CD16 clones on observed neutrophil PD-L1 expression in healthy young, healthy elderly and COPD participants. Neutrophils from (A) n = 6 healthy young (HY), (B) *n* = 4 healthy elderly (HE) and (C) n = 6 COPD participants were stained with anti-PD-L1 (BioLegend, Clone 29E.2A3) and co-stained with (+) or without (−) four different anti-CD16 antibodies: either different clones (i-ii, 3G8; iii, eBio-CB16; iv, VEP-13) or different manufacturers (i, Biolegend; ii, Invitrogen; iii, eBiosciences; iv, Miltenyi). Samples were analysed by flow cytometry, data expressed as median fluorescence intensity (MFI) per donor compared to single stained PD-L1 neutrophils. *P*-values determined using Wilcoxon matched-pairs signed rank test.

We therefore investigated if the increase in PD-L1 expression observed was specifically due to the epitope of anti-CD16 clone 3G8 (BioLegend) causing signalling induced by antibody ligation. To do this, we obtained three additional commercially available anti-CD16 antibodies. Neutrophils from healthy volunteers were incubated with anti-PD-L1 (BioLegend, clone 29E.2A3) and an equivalent concentration of each of the 4 different anti-CD16 antibodies (Table S2): the original AF700-conjugated, BioLegend, clone 3G8 (Fig. 2D); AF700-conjugated, Invitrogen, clone 3G8 (Fig. 3A ii); AF700-conjugated, eBioscience, Clone eBioCB16 (Fig. 3A iii); FITC-conjugated, Miltenyi Biotec, Clone VEP-13 (Fig. 3A iv). In each case, the presence of anti-CD16 caused the same observed increase in PD-L1 (Fig. 3A) with one exception – FITC-conjugated clone VEP-13 (Miltenyi).

Together, these data showed that the observed increase in PD-L1 surface expression was not dependant on antibody manufacturer and occurred in at least two independent clones of anti-CD16, but that at least one anti-CD16 antibody was capable of binding CD16 without causing changes in the observed PD-L1 expression (CD16 expression data in Supplementary Fig. S2).

### 3.5 Induction of PD-L1 in ageing and COPD

In order to assess if this effect was unique to healthy young donors, neutrophils from patients with COPD and age-matched healthy controls were also examined. Similar to neutrophils from healthy young volunteers, co-staining neutrophils from COPD patients (Fig. 3C) or age-matched controls (Fig. 3B) with AF700-conjugated anti-CD16 and anti-PD-L1 caused an increased observed PD-L1 expression, albeit not statistically significant in the healthy elderly population, likely due to sample numbers (Fig. C; Supplementary Table S2). Again, the notable exception was the FITC-conjugated anti-CD16 antibody (Miltenyi, clone VEP-13). Together, these data suggested that the effect was reproducible in both health, age and disease. The observed increase in PD-L1 was, therefore, either a fundamental biological phenomenon or was an artefact of the detection of PD-L1.

### 3.6 Fluorochrome specific PD-L1 induction

**Fig. 4:**
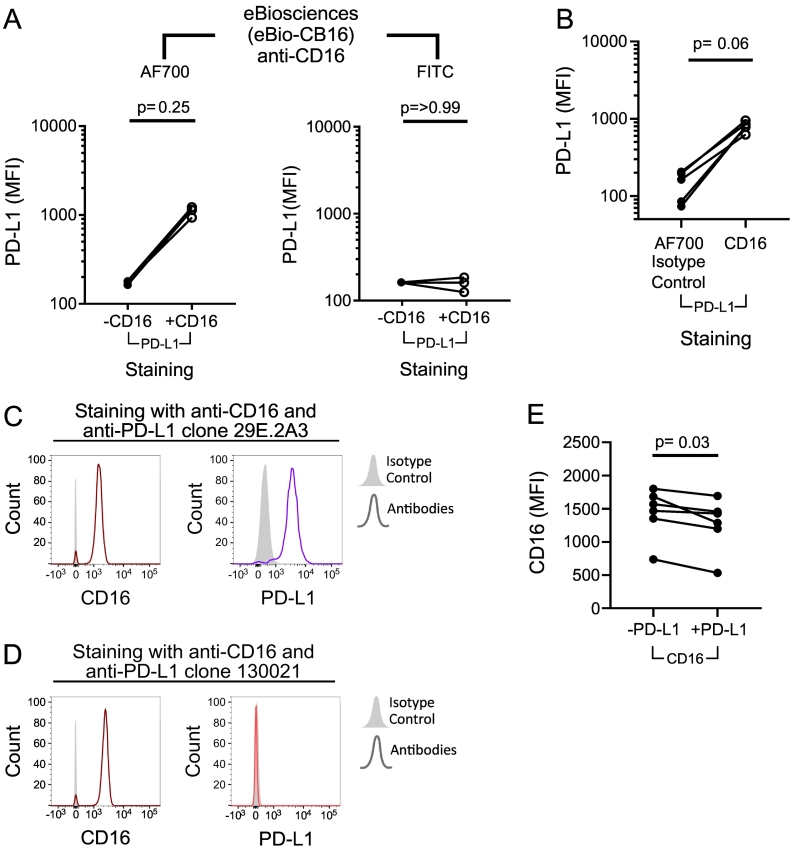
Investigation of alternative fluorophores of anti-CD16 and clones of anti-PD-L1 on the observed PD-L1 expression by human neutrophils. Neutrophils from healthy volunteers were analysed by flow cytometry and data expressed as median fluorescence intensity (MFI) per donor and *p*-values determined using Wilcoxon matched-pairs signed rank test in each instance. A) Neutrophils were stained with anti-PD-L1 (BioLegend; Clone 29E.2A3) and either AF700-conjugated or FITC-conjugated anti-CD16 (*n* = 3; eBiosciences; clone eBioCB16). B) Neutrophils were stained with anti-PD-L1 (BioLegend; Clone 29E.2A3) and either AF700-conjugated anti-CD16 (eBiosciences; clone eBioCB16) or an IgG isotype control (BioLegend; *n* = 5). C and D) Neutrophils from a single donor were stained with either anti-CD16 (eBiosciences; clone eBioCB16) and (C) anti-PD-L1 (BioLegend; Clone 29E.2A3) or (D) anti-PD-L1 (R&D Systems; clone 130,021) or an isotype control (filled grey). The MFI of both CD16 and PD-L1 are shown side-by-side. E) Neutrophils from n = 6 donors were stained with anti-CD16 (BioLegend; clone 3G8) with and without anti-PD-L1 (BioLegend; Clone 29E.2A3). The MFI of CD16 is shown on a linear scale.

In order to validate if the fluorochrome was responsible for this phenomenon, a FITC-conjugated anti-CD16 antibody (clone 3G8, Biolegend) was obtained as a direct comparison to the AF700-conjugated anti-CD16 antibody. Neutrophils obtained from healthy volunteers were stained with anti-PD-L1 (BioLegend, clone 29E.2A3) and either AF700-conjugated (as also shown in Fig. 3A ii) or FITC-conjugated anti-CD16 (eBiosciences, eBio-CB16; Fig. 4a). Only the AF700-conjugated anti-CD16 tended to produce the apparent increase in PD-L1 expression (Fig. 4A) as previously demonstrated in Fig. 3A ii. This is consistent with the lack of observed increase with FITC-conjugated anti-CD16 (Miltenyi, clone VEP-13) (Fig. 3A,B and C). Given these data, the apparent increase in PD-L1 expression by neutrophils appeared to be related to the AF700 fluorochrome and not specific binding of anti-CD16.

### 3.7 Novel mode of fluorochrome-binding antibody

In order to determine if the AF700 fluorophore was binding to neutrophils and causing an increase in PD-L1, we co-stained neutrophils with AF700-conjugated IgG1κ (BioLegend) and anti-PD-L1 (BioLegend, clone 29E.2A3) and observed a reduction in detected PD-L1 expression compared with neutrophils stained with AF700-conjugated anti-CD16 (Fig. 4B). It was, therefore, likely that the PD-L1 antibody was binding directly to the AF700 fluorochrome and there was no biological change in PD-L1 expression by neutrophils.

To test this, we purchased an alternative clone of anti-PD-L1 (Clone 130,021; R&D Systems, Abingdon, UK) and directly compared both anti-PD-L1 clones (clone 130,021, R&D Systems; clone 29E.2A3, BioLegend) on observed PD-L1 expression in conjunction with anti-CD16 (Clone eBioCB16, eBiosciences). As demonstrated previously, clone 29E.2A3 (BioLegend) resulted in detection of PD-L1 expression in combination with anti-CD16 (Fig. 4C). However, clone 130,021 (R&D Systems) did not reproduce this effect, allowing staining with AF700-conjugated anti-CD16 without an increase in the observed PD-L1 expression (Fig. 4D). Together, these data provide evidence of a novel interaction between clone 29E.2A3 of anti-PD-L1 (BioLegend) and the AF700 fluorochrome. This was supported by small decreases in the detected MFI of AF700 in the presence of anti-PD-L1 (clone 29E.2A3, BioLegend), likely due to disruption of the fluorescence signal from the direct binding of this antibody to the AF700 fluorophore (Fig. 4E).

**Fig. 5:**
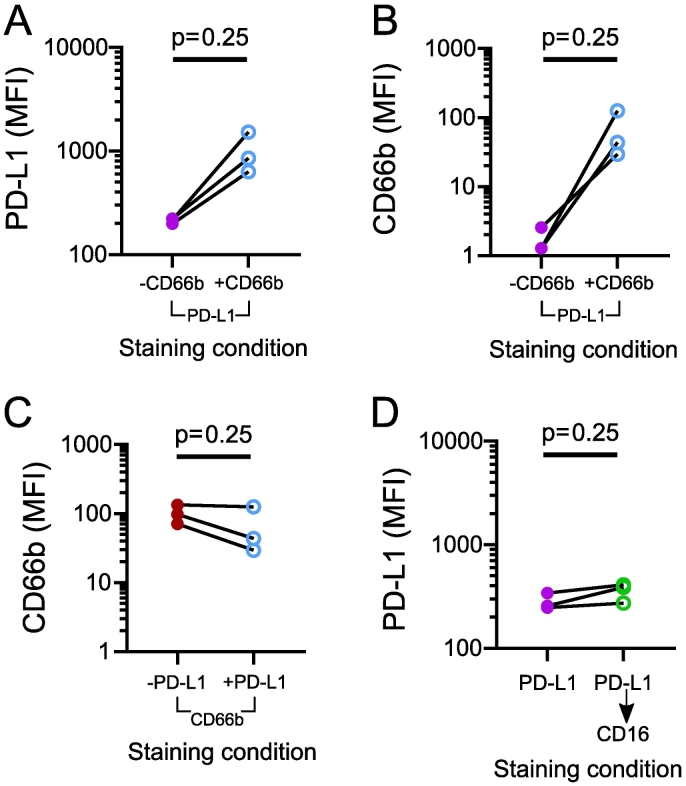
Median fluorescence intensity of PD-L1 and CD66b of neutrophils from healthy young participants incubated with anti-PD-L1 and AF700-conjugated anti-CD66b or anti-CD16. Isolated neutrophils from healthy young participants (n = 3) were incubated with combinations of anti-PD-L1 and anti-CD66 and the median fluorescence intensity (MFI) of (a) PD-L1 or (b) CD66b measured. (c) MFI of CD66b in the presence or absence of anti-PD-L1 antibody. (d) MFI of PD-L1 of neutrophils incubated with anti-PD-L1 and washed prior to incubation with anti-CD16. The median fluorescence intensity is shown on a log(10) scale for all plots.

Unfortunately, this clone of PD-L1 conjugated to BV605 was not commercially available to allow a direct clone comparison and, therefore, an alternative AF700-conjugated antibody was obtained (anti-CD66b [BioLegend]) to test the specificity of anti-PD-L1 to AF700. Neutrophils from healthy young volunteers were incubated with anti-PD-L1 and anti-CD66b either separately or together as previously for anti-CD16 antibodies. An increase in the detected PD-L1 expression was observed on neutrophils incubated with both antibodies compared with anti-PD-L1 alone (Fig. 5A and B). This increase in PD-L1 MFI was smaller than that observed with anti-CD16 antibodies, and is likely to be due to the lower abundance of the AF700 fluorophore, as demonstrated by the lower MFI recorded with anti-CD66b compared to anti-CD16.

Direct binding of this clone of anti-PD-L1 to AF700 was further suggested by a decrease in CD66b MFI observed upon the addition of anti-PD-L1 (Fig. 5C), as previously seen with anti-CD16 (Fig. 4E). Although, the decrease in CD66b MFI here did not reach statistical significance, probably due to a low sample number. Sequential staining with anti-PD-L1 and anti-CD16 (clone eBio-CB16) separated with a wash step, as noted in the manufacturer notes, failed to recapitulate the increase in PD-L1 as seen with co-incubation (Fig. 5D). Together, these results support the direct binding of anti-PD-L1 (clone 39E.2A3) to the AF700 fluorophore. It is also likely this interaction applies to any AF700-conjugated antibody.

## 4 Conclusion

Through careful validation of flow cytometry antibodies, we have shown that circulating neutrophils from healthy individuals and those with COPD show very low detectable levels of PD-L1 on their surface. Unlike in SLE, PD-L1 expression by neutrophils is unlikely to have a role in COPD, demonstrated in a study that did not use AF700-conjugated antibodies (Luo et al., 2016). Consequently, staining neutrophils with AF700-conjugated anti-CD16 led to false-positive detection of PD-L1 on the surface that initially appeared to be biological induction of PD-L1 expression through engagement of the CD16 receptor. We have demonstrated that the 29E.2A3 clone of anti-PD-L1 binds the AF700 fluorophore and therefore highlights a novel mode of cross-reactivity of this antibody – one that is likely to apply to other commercially used antibodies in specific combinations. It is, therefore, paramount that careful validation of antibodies used in multi-colour flow cytometry panels is carried out, especially when new combinations of clones, fluorophores and binding targets are used. Validation of antibody combinations in phenotyping antibody panels should become routine in publications to ensure that the discovery novel cellular phenotypes are accurate and subject to rigorous scientific review. The disclosure of appropriate controls, such as fluorescence minus one (FMO) controls (Maecker and Trotter, 2006), should also be included as these aid identification of false-positive results.

## Authorship

M.J.H. designed and performed experiments, analysed data, and wrote the original manuscript. H.M.M. and E.S. contributed to the study design and edited the manuscript. E.S. was principal investigator for recruited participants.

## Declaration of Competing Interest

ES has received funding from GSK. All other authors have no conflicts to disclose.

## Appendix A Supplementary data

Supplementary material Image 1
