# Supplementary material for: Importance of validating antibody panels: Anti-PD-L1 clone binds AF700 fluorophore
Source: J Immunol Methods. 2020 Aug;483:112795. doi: 10.1016/j.jim.2020.112795 (PMC7378575; doi:10.1016/j.jim.2020.112795)
Supplement: Supplementary file 1 — Supplementary material [file mmc1.docx]

**Importance of validating antibody panels: anti-PD-L1 clone binds AF700 fluorophore**

Michael J. Hughes^1,3^, Helen M. McGettrick^2^, Elizabeth Sapey^1^

^1^Birmingham Acute Care Research, Institute of Inflammation and Ageing, College of Medical and Dental Sciences, University of Birmingham, Birmingham, B15 2TT, UK.

^2^ Rheumatology Research Group, Institute of Inflammation and Ageing, University of Birmingham, Edgbaston, Birmingham, B15 2GW, UK

**Supplementary Data**

**
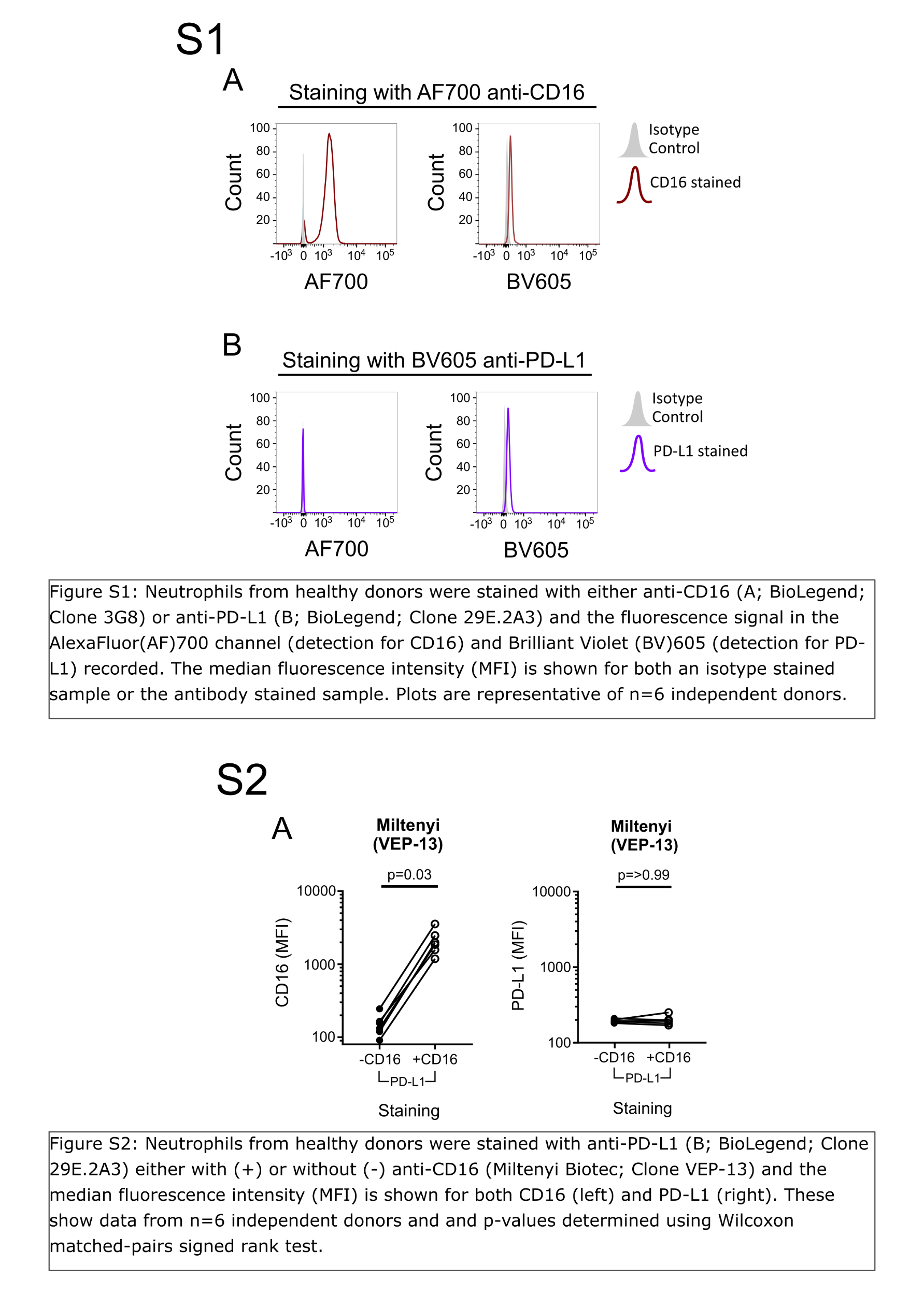
**

| **Supplementary Table S1:** Raw median fluorescence intensity data for each data point in Figure 2D | |
| --- | --- |
| **Condition** | |
| **PD-L1  -CD16** | **PD-L1  +CD16** |
| 183 | 7205 |
| 195 | 4831 |
| 211 | 9406 |
| 202 | 3253 |
| 191 | 4944 |
| 182 | 2810 |
| Due to the overlap of data points, the raw data for each of the 6 participants are presented here for clarity. | |

| **Supplementary Table S2:** Raw median fluorescence intensity data for each data point in Figure 3. | | | | | | | | |
| --- | --- | --- | --- | --- | --- | --- | --- | --- |
| **Figure** | **Condition** | | | | | | | |
| **3A - HY** | **i)** | | **ii)** | | **iii)** | | **iv)** | |
|  | **PD-L1 -CD16** | **PD-L1 +CD16** | **PD-L1 -CD16** | **PD-L1 +CD16** | **PD-L1 -CD16** | **PD-L1 +CD16** | **PD-L1 -CD16** | **PD-L1 +CD16** |
|  | 183 | 7205 | 183 | 2849 | 211 | 3046 | 183 | 202 |
|  | 195 | 4831 | 195 | 3034 | 202 | 1459 | 195 | 194 |
|  | 211 | 9406 | 211 | 4882 | 191 | 1405 | 211 | 198 |
|  | 202 | 3253 | 202 | 3345 | 162 | 1856 | 202 | 252 |
|  | 191 | 4944 | 191 | 1594 | 383 | 1526 | 191 | 181 |
|  | 182 | 2810 | 182 | 1225 | 182 | 1004 | 182 | 170 |
| **3B – HE** | **i)** | | **ii)** | | **iii)** | | **iv)** | |
|  | **PD-L1 -CD16** | **PD-L1 +CD16** | **PD-L1 -CD16** | **PD-L1 +CD16** | **PD-L1 -CD16** | **PD-L1 +CD16** | **PD-L1 -CD16** | **PD-L1 +CD16** |
|  | 207 | 10846 | 207 | 3671 | 207 | 2644 | 207 | 211 |
|  | 200 | 5462 | 200 | 2091 | 200 | 2676 | 200 | 192 |
|  | 183 | 5912 | 183 | 1069 | 183 | 1849 | 183 | 185 |
|  | 207 | 8244 | 207 | 4325 | 207 | 3273 | 207 | 209 |
| **3C - COPD** | **i)** | | **ii)** | | **iii)** | | **iv)** | |
|  | **PD-L1 -CD16** | **PD-L1 +CD16** | **PD-L1 -CD16** | **PD-L1 +CD16** | **PD-L1 -CD16** | **PD-L1 +CD16** | **PD-L1 -CD16** | **PD-L1 +CD16** |
|  | 194 | 14652 | 194 | 9664 | 194 | 4099 | 194 | 186 |
|  | 211 | 4435 | 211 | 2301 | 211 | 2044 | 211 | 195 |
|  | 183 | 5750 | 183 | 1683 | 183 | 1636 | 183 | 186 |
|  | 177 | 4682 | 177 | 1134 | 177 | 1791 | 177 | 173 |
|  | 188 | 5544 | 188 | 1920 | 188 | 1689 | 188 | 195 |
|  | 199 | 5568 | 199 | 1822 | 199 | 1906 | 199 | 207 |
| Due to the overlap of data points, the raw data for each of the participants in each group (HY: healthy young; HE: healthy age-matched; COPD: patients with chronic obstructive pulmonary disease) are presented here for clarity. | | | | | | | | |
